# Mr. Morgan's Cases of Vaccination

**Published:** 1807-04

**Authors:** J. Morgan

**Affiliations:** Surgeon. Ipswich


### *To the Editors of the Medical and Physical Journal.*

Gentlemen,

At a time like the present, when Vaccination is undergoing the most strict investigation, I conceive it to be the duty of every practitioner, possessed of useful information, to bring it forward. Among the many facts which will ever establish its excellence, is that of infants successfully vaccinated at the breasts of mothers or nurses labouring under small pox.

In January last, Mrs. Finch, of Otley, in this neighbourhood, had small-pox of the distinct kind. I saw her on the sixth day of the eruption, when she had a healthy child five months old at the breast, but unaffected by the mother's complaint. I instantly vaccinated the child, who went through the cow-pox.

About the same period, I was called to another instance of variola, in Mrs. Woolner, of Grundisburgh, six miles from this place, who had a child eight months old at her breast. The pustules were less distinct than in Mrs. Finch, but more numerous. On the fifth day of the eruption I vaccinated the child, and succeeded most completely, and without difficulty, in preventing small-pox.

In both these Cases I advised a removal of the children from their mothers; but my request was not complied with, for the babes continued at the breast without experiencing the slightest inconvenience.

I am, &c.

*Ipswich, March* 10, 1807. J. MORGAN, Surgeon.

